# Modeling Varicella Zoster Virus Persistence and Reactivation – Closer to Resolving a Perplexing Persistent State

**DOI:** 10.3389/fmicb.2019.01634

**Published:** 2019-07-24

**Authors:** Lillian Laemmle, Ronald S. Goldstein, Paul R. Kinchington

**Affiliations:** ^1^Department of Ophthalmology, University of Pittsburgh, Pittsburgh, PA, United States; ^2^Faculty of Life Sciences, Bar Ilan University, Ramat Gan, Israel; ^3^Department of Molecular Microbiology and Genetics, University of Pittsburgh, Pittsburgh, PA, United States

**Keywords:** varicella zoster virus, latency, reactivation, animal models, human neuron culture models

## Abstract

The latent state of the human herpesvirus varicella zoster virus (VZV) has remained enigmatic and controversial. While it is well substantiated that VZV persistence is established in neurons after the primary infection (varicella or chickenpox), we know little of the types of neurons harboring latent virus genomes, if all can potentially reactivate, what exactly drives the reactivation process, and the role of immunity in the control of latency. Viral gene expression during latency has been particularly difficult to resolve, although very recent advances indicate that it is more restrictive than was once thought. We do not yet understand how genes expressed in latency function in the maintenance and reactivation processes. Model systems of latency are needed to pursue these questions. This has been especially challenging for VZV because the development of *in vivo* models of VZV infection has proven difficult. Given that up to one third of the population will clinically reactivate VZV to develop herpes zoster (shingles) and suffer from its common long term problematic sequelae, there is still a need for both *in vivo* and *in vitro* model systems. This review will summarize the evolution of models of VZV persistence and address insights that have arisen from the establishment of new *in vitro* human neuron culture systems that not only harbor a latent state, but permit experimental reactivation and renewed virus production. These models will be discussed in light of the recent data gleaned from the study of VZV latency in human cadaver ganglia.

## Introduction

Varicella zoster virus (VZV) is a common and ubiquitous human-restricted neurotropic alphaherpesvirus of the *Herpesviridae* family that persists for life in the host after a primary infection (varicella or chickenpox). The site of latency is within neurons in ganglia of the peripheral somatic, autonomic, and enteric (Gershon and Gershon, 2018) nervous systems. Up to one third of infected individuals will clinically reactivate VZV in their lifetimes, usually in their elderly years when immunity is naturally senescing, or when immunity is suppressed by disease or iatrogenic cause. The most common clinical sign of reactivation is herpes zoster (HZ), manifested as a dermatome-limited, painful vesicular skin rash that causes greater morbidity than varicella with frequent complications. VZV reactivation may also underlie a variety of neurological (Gilden et al., 2015), vascular (Nagel et al., 2017), and gastrointestinal diseases (Gershon and Gershon, 2018) that may occur with or without rash.

A greater understanding of the events occurring during VZV latency and early reactivation may reveal targets and strategies for novel therapeutics that treat or prevent HZ. However, VZV shows a high degree of host specificity, which has precluded most small animal modeling of VZV disease. There is arguably still no *in vivo* model of reactivated disease. A possible exception for the growth restriction of VZV in small animals is the guinea pig, discussed below. Many insights have come from study of the VZV-related Simian Varicella Virus (SVV), which replicates in several macaque species and African green monkeys, but SVV is a distinct virus that may not completely model all aspects of VZV. There are also significant financial and ethical issues associated with primate research. The relevance of the SVV model to VZV was recently reviewed (Sorel and Messaoudi, 2018).

A second issue that has made VZV persistence difficult to understand is the difficulty in interpreting studies of VZV-infected human cadaveric ganglia. We are now more cognizant that a partial viral gene expression program occurs throughout the post mortem interval (PMI) (Cohrs and Gilden, 2003; Ouwendijk et al., 2012). Early reports suggesting that VZV proteins were made during latency were subsequently complicated by staining artifacts or antibody cross-reactivity to blood group A1 antigens (Zerboni et al., 2012). Very recent work indicates VZV latent transcription is highly restricted in ganglia obtained with short PMI (Depledge et al., 2018a). Even with short PMI, cadaver ganglia rapidly undergo hypoxia that may induce death-induced spurious transcription. Importantly, we are not aware of any report of VZV virus production obtained from latently infected cadaveric ganglia explants. This contrasts to HSV-1, which readily produces virus from human axotomized ganglia (Lewis et al., 1982).

A third block to study of VZV latency is that, despite the efforts of many virologists, VZV remains a difficult virus to work with. Virus replicates inefficiently in a limited number of human cell lines, produces virus with high particle to infectivity ratios, and releases little to no infectious virus from cultured cells. Titers of cell-free virus released from infected cell sonicates are on the order of five magnitudes lower than that which can be obtained for HSV-1. As such, most experimental infections are initiated with infected cells. As detailed below, this is an important issue in the new stem cell-derived neuronal models of reactivatable latency, because persistence has not yet been obtained with cell-associated infection, but rather requires cell-free virus. Intriguingly, recent work suggests that cultured human neurons do release some infectious virus (Markus et al., 2011; Sadaoka et al., 2018).

This review complements other recent reviews of the current state of understanding of VZV latency from studies of cadaver ganglia (Depledge et al., 2018b) and *in vitro* models (Baird et al., 2019). We restrict this review to human VZV and discuss the potential of models that may be revisited in the near future, particularly in light of recent discoveries.

## Human VZV Disease

Primary varicella (chicken pox) is easily spread in unvaccinated Westernized populations, usually during childhood, by the inhalation of aerosolized droplets from individuals with varicella or active zoster. This exposure establishes primary replication sites in the upper respiratory mucosa and respiratory lymphoid tissues such as the tonsils, where lymphocytes become infected (Ku et al., 2005). VZV infected CD4^+^ T cells are considered to be a predominant vehicle for systemic spread (Ku et al., 2005), but other circulating cells have been implicated in the viremia, including dendritic cells, monocytes and macrophages (Kennedy et al., 2019). Immune cells carrying VZV home to the skin and extravasate, seeding sites of virus infection at the deep dermal skin layers and hair follicles (Ku et al., 2002). Virus accesses peripheral innervating ganglia by infecting axons in the skin, followed by retrograde transport down axons to neuronal soma. Evidence from the SVV model suggests that ganglia are also directly infected by ganglion-infiltrating VZV-infected T cells at the viremic stage (Sorel and Messaoudi, 2018), allowing infection of ganglia that do not necessarily innervate the periphery, such as those of the enteric nervous system (Ku et al., 2002; Chen et al., 2003, 2011; Gershon et al., 2008). In the 14–21 days incubation period, VZV replication in the skin is partly controlled by innate immune mechanisms, including type 1 IFNs (Zerboni et al., 2014). Lesions then develop in the epidermis, concurrent with the onset of adaptive immunity that not only clears virus and infected tissues, but also protects against further varicella disease.

Reactivation leading to HZ occurs in about one third of those harboring latent VZV in their lifetimes. While the molecular triggers of VZV reactivation are not well understood, there are clear contributing factors that predispose to HZ development, including host genetics (Crosslin et al., 2015). The role of immunity in reactivation was established by the classic studies of Hope-Simpson (Hope-Simpson, 1967), and is evidenced by the fact that HZ rates are higher in those with immune modifying diseases such as HIV infection, or undergoing treatments that reduce cellular immune function. HZ incidence rises rapidly after age 60 years, and this may partially reflect a naturally senescing cellular immunity (Donahue et al., 1995). Clinical zoster probably requires both intracellular events in the host neuron that influence viral chromatin, as well as a failure in surveillance/protection by VZV-specific cellular immunity. The large lesions of HZ are clinically distinct from varicella in that they are usually topographically restricted to a skin region innervated by a single ganglion, termed a dermatome. The extensive lesions reflect the intra-ganglionic spread of virus after reactivation and the delivery to the skin by multiple neurons in a reactivating ganglion. It is not yet clear if reactivation originates from a single neuron or from multiple neurons triggered in parallel, nor is it clear if intra-ganglionic spread triggers sequential reactivation from other latently infected neurons in the same ganglia.

Zoster is frequently complicated by scarring and bacterial superinfection, but the most common complication is pain. Most individuals with zoster seek medication for pain, but a significant fraction progress to debilitating chronic pain states termed post herpetic neuralgia (PHN). PHN may last months to years after the skin lesions have resolved and is often refractory to therapy. This is consistent with PHN reflecting neuronal events downstream of VZV replication and clearance after reactivation (Gershon, 1996). Reactivation causes massive inflammatory responses in the ganglia that may change neuron function, pain signaling and neuronal activity, but these transitions are poorly understood. A review of neurobiological models of PHN was recently published (Devor, 2018).

## Vaccination

The observation that adaptive immunity protects individuals from further varicella stimulated the development of successful vaccines. The FDA-approved live attenuated varicella vaccine (Varivax in the United States) has been in widespread use in the United States since the mid-1990s as a two-dose regimen. It is based on a VZV strain (vOka) that was attenuated by sequential passage in embryonic guinea pig fibroblasts. The vaccine strain is genetically heterogeneous, containing some fixed mutations and variable levels of other mutations and combinations thereof (Depledge et al., 2014; Quinlivan and Breuer, 2014). Varivax is administered to most United States preschool children and has dramatically reduced the number of varicella cases in the United States, as well as hospitalizations and deaths from it. However, many countries do not widely use the varicella vaccine, and even in the United States, most adults still harbor wild-type VZV with the potential to reactivate. The first individuals who received Varivax will not turn 60 years, the age when HZ incidence rises, until around 2050. The live vaccine retains the ability to establish latency and reactivate, albeit at very low levels (Hardy et al., 1991; Quinlivan et al., 2012).

Two FDA-approved HZ vaccines are available to boost VZV-specific adaptive immunity in those at risk for HZ. Zostavax is a 14 times larger infectious dose of the attenuated varicella vaccine that was licensed in 2006, and reduces HZ incidence by about half, and the burden of illness by two thirds (Oxman et al., 2005). The more recent Shingrix vaccine was FDA licensed in 2018 and is a subunit vaccine based on the VZV gE glycoprotein. It has higher efficacy than Zostavax in preventing HZ using a two-dose regimen (Cunningham et al., 2016). Both vaccines are under-utilized in the target population due to perceived lack of efficacy, cost, ignorance of the target population to HZ severity, and vaccine shortages (Harpaz and Leung, 2017).

## Definition of the VZV Latent State

We consider it important to define “latency.” Herpesvirus lytic infections involve the organized and regulated expression of most viral genes and proteins, the replication of the DNA genome, and the assembly and egress of progeny virus for spread and transmission. VZV latency is often defined as the persistence of viral genomes without virus production, in an endless or episomal form (Clarke et al., 1995) as seen for the latent state of all other herpesviruses. However, this definition may be inadequate: it does not separate reactivatable latency from an abortive infection in which genomes are maintained (perhaps due to a feature of the host cell, the neuron) that cannot complete a lytic cycle due to absence of factors required for full lytic growth. Human VZV latency is perhaps a mixture of both states as the vast majority of maintained genomes will never undergo a reactivation event. This scenario was highlighted in studies on the murine neuronal model of HSV-1 latency, where hundreds to thousands of genomes are maintained in multiple types of neurons, including some subtypes that do not appear to be capable of supporting productive infection (Bertke et al., 2011). We highlight this issue because most small animal models do not appear to support full VZV replication. Are the genomes present in such models stalled by the non-permissive state, and is this different from that in humans? We propose latency should be defined as the *reversible* persistence of non-productive viral genomes, since reactivation demonstrates maintained genome functionality.

A second issue is the silencing of genome expression during latency. Are viral RNAs or proteins made during the latent state? This question is of considerable interest, because any viral product made during latency could be potentially targeted by therapeutic strategies. Again, we turn to the more extensively studied HSV-1 as a guide. In HSV latency, expression is predominantly silenced by chromatin on the genome (Bloom et al., 2010). The exception is the non-coding transcripts termed Latency-Associated Transcripts or LAT (see below). However, HSV-1 latency is not necessarily antigenically silent, because latently infected ganglia in animal models (Knickelbein et al., 2008) and in humans (van Velzen et al., 2013) are associated with recently activated virus-specific CD8^+^ T cells. In the well characterized HSV-1 murine model, accumulated work suggests that ganglionic T cells may control reactivation events after viral antigen recognition, acting through non-cytolytic mechanisms (Decman et al., 2005; Knickelbein et al., 2008). Such antigens arise through a dynamic state of repression during latency. An attractive model has proposed that HSV-1 reactivation involves a two-stage de-repression process, with the first stage being reversible to the silent latent state (Kim et al., 2012). The initial de-repression is driven by reactivation stimuli that alter chromatin states and drive a transient expression of viral proteins outside the typical lytic cascade. This state may or may not progress to a typical lytic-regulated cascade and virus production, or may reverse to quiescence, depending on the functional expression and cellular localization of viral factors initiating the lytic cascade. This two-step model of de-repression fits with the proposed role of ganglion-resident T cells in monitoring gene expression in the ganglia. Resident T-cells would be rapidly activated in response to renewed viral protein expression, and act using non-cytotoxic mechanisms that favor re-entry into latency (St Leger and Hendricks, 2011).

For VZV, a new picture is emerging that suggests that VZV gene expression during human latency is more limited than was once thought (Cohrs and Gilden, 2003; Ouwendijk et al., 2012; Zerboni et al., 2012). Using unbiased Illumina next generation sequencing methods coupled with Agilent’s SureSelect template enrichment of VZV RNA from human ganglia obtained with short PMI times, only two VZV transcripts were detected (Depledge et al., 2018a). Low levels of mRNA from ORF63 were observed confirming previous reports, but a more abundant transcript termed VZV Latency-associated Transcript (VLT) was mapped to the VZV region encoding ORF61, but from the antisense strand. This extensively spliced transcript extends into and past ORF61, a gene that encodes a key transcriptional regulator with E3 ubiquitin ligase activities (Wang et al., 2011; Zhu et al., 2011). The authors proposed that VLT works in an antisense manner to regulate ORF61, but it is not clear if this occurs in the context of VZV infection or latency. However, the VLT genomic position and direction are conserved with the latency-associated transcripts made by SVV, HSV-1, bovine herpesvirus type 1, and indeed, perhaps all neuronal alphaherpesviruses, raising the interesting concept of conserved functionality as these viruses have diverged. VZV VLT spliced RNA potentially encodes a short protein, a feature that is not fully conserved in these viruses; for example, while the vLAT of bovine herpesvirus encodes a protein, HSV-1 LAT does not. However, HSV LAT does encode miRNAs (Umbach et al., 2008). Intriguingly, two small non-coding RNAs found in VZV-infected cells, snc13 and snc14, map to the unspliced VLT RNA (Markus et al., 2017). It has been suggested that VZV ORF63 protein regulates apoptosis in neurons (Hood et al., 2006), an activity attributed to both the HSV LAT RNA and the BHV LAT protein (Jones, 2013). While these are exciting findings, we argue that some caution is still needed. Even a short term post mortem event cannot be avoided in human studies, and we do not know if all persisting VZV genomes express these transcripts. A similar transcript found in macaques latently infected with SVV with no PMI suggests VLT is authentic (Sorel and Messaoudi, 2018). Many questions still remain. Are VZV VLT and/or ORF63 RNA functionally involved in the establishment and/or maintenance of VZV latency? If so, how do they function? Is VLT made in animal models harboring VZV genomes? Are these RNAs made in all neurons hosting a VZV genome? We argue that a solid model of latency and reactivation is needed to address these issues.

## *In Vivo* Models of VZV Latency

No small animal model recapitulates human disease, even after administration of high levels of VZV infectious units. Animals often seroconvert, but this is not surprising given the antigen in the cell-associated inoculates. Here, we discuss attempts at developing a model of VZV persistence and discuss those that may be re-evaluated in light of the new picture of human VZV latency transcription (summarized in Table 1).

**TABLE 1:** Permissivity of currently available *in vivo* and *in vitro* systems for modeling VZV infection, latency, and reactivation.

**Model**	**DNA in infected tissue**	**DNA in neurons/ganglia**	**RNA**	**Protein**	**Replication**	**Spread**	**Latency/Quiescence**	**Reactivation**
***In vivo***								
Mice	✓	✓						
Rat	✓	✓	✓					
Cotton rat	✓	✓	✓					
Guinea pig^*^	✓	✓	✓	✓	✓	✓	✓	?
SCID-hu mouse/human xenograft^†^	✓	✓	✓	✓	✓	✓	✓	
***In vitro***								
Rat primary/progenitor cells	✓	✓	✓					
Immortalized human neuron-like cells	✓	✓	✓	✓	✓	✓		
Differentiated human neural progenitor lines	✓	✓	✓	✓	✓	✓		
hiPSC-derived neurons	✓	✓	✓	✓	✓	✓		
hESC-derived neurons	✓	✓	✓	✓	✓	✓	✓	✓

While many models support VZV infection and limited viral RNA and protein expression programs, VZV replication and spread are limited to guinea pigs and human cells that are permissive for the full lytic virus life cycle. hESC-derived neurons are the only model in which experimental reactivation of a quiescent VZV infection has been reliably achieved. It has been claimed that the guinea pig model supports experimental reactivation. ^*^Most studies use guinea pig adapted VZV. ^†^In human tissue graft.

### Mice and Rats

Immunocompetent mice have not been widely used to study VZV latency and evidence indicates they do not support VZV replication. VZV introduced to the cornea by intrastromal placement of infected cells resulted in virus DNA reaching the TG (Wroblewska et al., 1993) but no clinical sign of infection was seen. VZV DNA in nervous tissues was seen by PCR at 33 days post infection. The refractory nature of the mouse to VZV suggests an abortive infection, where VZV enters axons at the site of inoculation and undergoes retrograde transport to the ganglia but is unable to complete the full lytic cycle. Restriction of VZV replication was also seen in Chinese hamster cell lines (Finnen et al., 2006) and rat primary cultures (Guedon et al., 2015). Severely compromised immune deficient mice show no evidence of VZV replication except in implanted human tissue (Moffat et al., 1998).

A similar situation appears to occur in rats. Multiple subcutaneous inoculations of VZV-infected human cells along the rat spine (Sadzot-Delvaux et al., 1990, 1995) did not lead to any clinical signs out to 9 months, but VZV DNA was detected in ganglia. No infectious virus was recovered from dissociated DRG cultures, although they did report live virus recovery after multiple rounds of ganglionic co-culture with VZV permissive MRC5 cells. Other groups have seen VZV DNA and, in some cases, RNA in rat ganglia corresponding to infected sites (Annunziato et al., 1998; Kennedy et al., 2001). The Sadzot-Delvaux group were the first to suggest ORF63 as a “latency associated gene” (Debrus et al., 1995). Brunell et al. (1999) studied VZV inoculated neonatal rats and detected RNA for the once-proposed “latency associated gene” from ORF21. It appears that these models have not been further developed.

Some attention was given to the outbred cotton rat, since it is more permissive for several human-specific viruses (Faith et al., 1997; Niewiesk et al., 1997; Langley et al., 1998). VZV was reported to replicate in cotton rat fibroblast cultures, but it has never been clear if VZV replicates *in vivo*. Cell-associated VZV inoculated by intramuscular or subcutaneous routes along the spine does lead to detectable levels of VZV DNA at ganglia and expression of ORF63 RNA. The model was extensively developed to assess the genetic requirements and ganglionic delivery of viral DNA, including several genes that are considered essential or near critical for lytic replication of VZV (Sato et al., 2002a). VZV genes deemed not to be required for DNA delivery to the ganglia included ORF61 (Sato et al., 2002b) and ORF17 (Sato et al., 2002a), both of which severely impair VZV growth when deleted. Surprisingly, DNA from VZV deleted for the essential ORF21 (Xia et al., 2003) was still delivered to ganglia. However, in these studies, the virus was inoculated in the complementing cells, so that functional virus was produced and able to infect axons for ganglionic delivery. In contrast, VZV mutants lacking the essential genes ORF63, ORF4 and ORF29 genes were considered deficient for ganglionic delivery of viral DNA in this model (Cohen et al., 2005a, b, 2007), but viruses were passaged once in non-complementing cells before inoculation. The mechanisms underlying these results are not clear, but fit with a model that as long as VZV is produced in the cell-associated inoculum, it can infect axons and reach the ganglia. The single pass of VZV in non-complementing cells before inoculation for the ORF63, 4 and 29 mutant studies would have resulted in a stalled infection and no virus production. Only one study did not fit this model, and reported that several recombinant VZV with alterations of portions of the ORF62 and ORF63 genes and the 3′ genome region could replicate in culture, but were deficient in ganglionic delivery of genomes (Ambagala et al., 2010).

We highlight that neither *in vivo* replication nor reactivation in the cotton rat has been demonstrated. The maintenance of viral DNA in rat ganglia is consistent with VZV entry into rat cells (and neuronal axons) without need for complete replication (Guedon et al., 2015). It will be interesting to determine if VLT is made in rat models, particularly since Wistar and Sprague Dawley rats develop prolonged behavioral signs of VZV-induced mechanical hypersensitivity (Fleetwood-Walker et al., 1999; Dalziel et al., 2004; Garry et al., 2005; Hasnie et al., 2007; Guedon et al., 2014, 2015; Kramer et al., 2017; Stinson et al., 2017).

### Guinea Pig

The guinea pig appears different from rats and mice because it can support a productive VZV infection, particularly with VZV adapted by passage in primary guinea pig embryo or kidney fibroblast cultures. Guinea pig cells are known to be one of the few non-primate lines to support VZV replication, and the vaccine (vOka) was attenuated by serial passaging in guinea pig embryonic fibroblasts. The genetic changes in vOka that are associated with adaption are many, including several in the gene encoding IE62, the key *trans*-activator of gene expression (Wu et al., 2019). Early work reported VZV infection of both adult and weanling guinea pigs (Myers et al., 1980, 1985, 1991; Lowry et al., 1993), resulting in a viremia and seroconversion after nasal inoculation (Matsunaga et al., 1982). Viral transmission to cohoused animals based on seroconversion was also indicated, but animals did not exhibit observable clinical symptoms (Myers et al., 1980, 1985). The guinea pig model was used extensively in the early characterization of the cellular immune response to VZV (Jenski and Myers, 1987) and the predominant viral antigenic targets (Lowry et al., 1992, 1993, 1997; Sabella et al., 1993). After infection, VZV DNA is detected in ganglionic tissues, but evidence of *in vivo* reactivation was lacking, even after treatment with immunosuppression regimens. One report indicates that VZV in newborn hairless guinea pigs induced a short-term skin exanthem (Myers et al., 1991). The guinea pig was also developed as an ocular infection model. Following intrastromal inoculation, VZV could be recovered from TG, midbrain, and cerebellum by co-culture with permissive cells (Pavan-Langston and Dunkel, 1989). Others have found no signs of disease or infection after corneal inoculation (Matsunaga et al., 1982).

There is renewed interest in the guinea pig for modeling VZV infection and persistence in the enteric nervous system. Enteric forms of zoster in humans have now been observed (Pui et al., 2001; Chen et al., 2003, 2011; Edelman et al., 2010; Masood et al., 2015; Gershon and Gershon, 2018). Cultures comprised mostly of enteric primary afferent neurons derived from adult hosts can support a lytic VZV infection after cell-associated inoculation, yet retained VZV DNA long term after cell-free virus infection. However, a rather wide gene expression pattern was reported, with expression of multiple viral genes during persistence, including from ORFs 4, 21, 29, 40, 62, and 63. This gene expression is more extensive than seen in human cadaver ganglia, and occurs presumably with no PMI. Some protein expression was reported as “mislocated” in the cytoplasm (Chen et al., 2003; Gershon et al., 2008). However, long term VZV DNA-positive *in vitro* neuron cultures could produce infectious virus when infected with replication defective adenoviruses expressing VZV ORF61 or the HSV ortholog ICP0. This establishes functionality of the persisting genome, albeit using a non-physiological stimulus. The model has developed in parallel with data indicating VZV persistence in the human enteric nervous system (Gershon and Gershon, 2018). Resected intestinal tissues from VZV seropositive and negative children, or of vaccine recipients, contained VZV DNA and expressed VZV transcripts (Chen et al., 2011). In the guinea pig model, intravenously injected T-lymphocytes infected *in vitro* with VZV were found to transmit viral DNA to multiple neurological tissues, including the DRG and enteric nervous system (Gan et al., 2014). VZV was reported to be experimentally reactivated by administration of tacrolimus in combination with corticotrophin-releasing 4 hormone to mimic stress, but important experimental data was not shown (Gershon and Gershon, 2018). Taken together, the recent work suggests the guinea pig is the strongest of the *in vivo* small animal models for study of VZV latency *in vivo*, and may be experimentally reactivatable. More work is needed to optimize reactivation strategies and establish similarities to human VZV persistence.

### Human Tissues in SCID Mice

SCID mice are unable to reject implanted human tissues (the SCID-hu model) and have proven to be a powerful tool for *in vivo* studies of human specific viruses, albeit in the absence of any adaptive immunity. Implanted tissues retain many characteristics and structural organization for long periods in these mice, despite an unnatural ectopic location (McCune et al., 1988; Mosier et al., 1988). The application of the SCID-hu mice for VZV was pioneered by Moffat et al. (1995), using skin tissue from fetuses and, more recently, adults (Zerboni et al., 2018). Immune cells from grafted human fetal thymus and liver tissue are placed beneath the mouse kidney capsule, as are fetal ganglia. A wealth of important information on VZV pathogenesis patterns has been gleaned from these models, and the roles of many viral genes in specific tissue infections have been elucidated (Zerboni et al., 2014). Since we are addressing models of latency, we will restrict our review to the use of human fetal DRGs in the SCID-hu system.

Fetal implants of intact ganglia in SCID-hu mice maintain much of the structural organization seen in ganglia *in situ*, despite axotomy during acquisition. Neurons are surrounded by ganglionic glia and satellite cells, in a physiological state that permits glial cell–neuron interactions in VZV pathogenesis to be followed (Hanani, 2005; Zerboni et al., 2005). DRG neurons express many of the expected markers, synaptophysin and neural cell adhesion molecule (NCAM), and by 4 weeks post implantation contain differentiated subsets of nociceptive and mechanoreceptive neurons marked by peripherin and RT97 antibodies respectively. VZV infection can be tracked by tissue extraction and subsequent processing of the graft for presence of virus and IHC, or in real time by using VZV expressing firefly luciferase from viral promoters coupled with IVIS bioluminescent imaging (Jones et al., 2006; Oliver et al., 2008). VZV inoculated directly into grafts showed increasing replication during the first 2 weeks, followed by a decline to control levels by 4–6 weeks post infection. DRG xenografts could also be infected by tail vein injection of VZV-infected human T-lymphocytes, supporting the direct viremia infection of ganglia that is speculated to occur in human varicella. Acute infection is accompanied by extensive cytopathology that is similar to that seen in human ganglia of patients dying with active zoster in that there is loss of cellular integrity, fusion events between multiple cell types including neurons and support cells, the production of virus, and changes in the inflammatory state and cytokine milieu of the ganglia. The diminishment of the acute ganglionic replication and its resolution suggests there is no need for adaptive immunity for the establishment of persistence and the cessation of ganglionic lytic replication (Schaap et al., 2005). VZV genomes persisted at low levels for months in the xenograft and were interpreted as a transition to a latent infection. A recent correlation of infection with neuronal markers suggest that mechanoreceptor neurons marked by RT97 antibodies are more resistent to VZV replication (Zerboni and Arvin, 2011). It is not clear if these give rise to reactivatable latent infections or are more reflective of a cell restricted or abortive infection. Ganglia contained low levels of transcripts of ORF63 but showed little VZV protein expression (Pevenstein et al., 1999; Zerboni et al., 2007; Zerboni and Arvin, 2011). Intriguingly, it was demonstrated that the vaccine Oka strain retained the ability to establish a persistent state in SCID-hu DRG (Zerboni et al., 2005), supporting that the vaccine retains the ability to efficiently establish latency. A second intriguing observation was that persistence was not established if the interactions of the gI and gE glycoproteins were genetically interrupted. Rather VZV entered into a chronic low replication phase maintained over time, suggesting that this critical interaction somehow influences lytic/latent decisions of the virus (Zerboni and Arvin, 2011).

To date, spontaneous or experimentally induced reactivation from the persistent state has not been reported in the SCID-hu DRG model so it is not yet clear whether the persistent infection is latent, abortive or a mixture of both. While clearly this is a solid model for study of VZV neurotropism, additional work is required to establish if maintained VZV genomes are reactivatable.

## *In Vitro* Models of VZV Latency and Reactivation

### Non-stem Cell Derived Neuron Cultures

There have been several attempts to develop *in vitro* systems for study of the VZV latent state (summarized in Table 1), and most have focused on human cultured neuron systems that should overcome the species restriction of VZV. Attempts using cultures derived from rat primary ganglia, rat embryonic ganglia and rat neuron progenitor lines resulted in the expression of a limited viral gene program, which nevertheless could alter host expression patterns (Kennedy et al., 2001, 2013; Hamza et al., 2007) and apoptosis (Hood et al., 2006). The lack of full viral replication is consistent with the observations of Guedon et al. (2015) that the rat does not support full VZV replication.

*In vitro* systems using cultured neurons from human adult cadaver ganglia are largely unsuitable for experimental infections leading to persistence because most are likely to harbor latent VZV and/or HSV. The latter has the potential to initiate a full productive reactivation (Bastian et al., 1972; Baringer and Swoveland, 1973; Lewis et al., 1982; Warren et al., 1982). However, analyses of cadaver ganglia were critical in establishing the presence of VLT and ORF63 transcripts during latency in humans (Depledge et al., 2018a). Reactivation of endogenous VZV from intact or dissociated ganglionic cultures leading to virus production has not been achieved (Azarkh et al., 2012), but axotomized cadaver ganglia show increased VZV DNA production upon the interruption of NGF signaling (Cohrs et al., 2017). NGF signaling is required to maintain HSV latency in murine and rat experimental neuron systems (Wilcox and Johnson, 1987; Camarena et al., 2010). Axotomized ganglia obtained from aborted fetuses offer an alternative as they are unlikely to contain pre-existing herpesvirus latent genomes. However, their use raises ethical issues and presents logistical difficulties in obtaining fresh neuronal tissue that survives propagation in culture. The inherent genetic variability between donors is also potentially problematic. In addition to the studies of VZV infection of fetal ganglionic xenografts in SCID mice just described, intact and dissociated cultured human fetal ganglia *in vitro* has been shown to host a productive experimental VZV infection (Hood et al., 2006; Gowrishankar et al., 2007; Steain et al., 2011). The use of fetal tissues in modeling VZV latency and reactivation *in vitro* has not yet been reported.

Several attempts have been made to exploit neuron-like cells differentiated from cancer or immortalized lines for study of VZV, particularly the human neuroblastoma cell line SH-SY5Y. Both SH-SY5Y cells and their differentiated neuron-like derivatives can host full VZV replication and intracellular virus spread (Christensen et al., 2011). While they offer the advantage of expandability, they suffer the disadvantage of generating terminally differentiated cells from transformed lines that do not have all the characteristics of adult human ganglionic neurons. SH-SY5Y have been widely used with HSV and drug-induced genome persistence (Shipley et al., 2017), but a reactivatable VZV latent state has not been reported. The repertoire of other expandable cell lines continues to grow and awaits evaluation for modeling VZV latency.

Human neurons can be also differentiated from commercially available neural progenitor cells. Pugazhenthi et al. (2011) exploited commercially obtained neurospheres derived from human fetal brain to generate neuron cultures to host a VZV infection. The cultures were 90% positive for the early neuronal markers MAP2a and βIII tubulin, and expressed multiple VZV transcripts and proteins after infection, intriguingly without cytopathic effect. It was not established whether a full productive VZV infection was supported, and homogenates of infected cultures could not initiate infection of VZV-permissive fibroblasts, suggesting that this may not be a sufficiently robust model for exploring VZV latency.

Goodwin et al. (2013) detailed the use of stem cell-derived neuroprogenitors from the brain to derive long term cultured 3D assemblies containing neurons surviving for up to 6 months. The 3D systems could host a spreading VZV infection and maintain viral genomes long-term, with sporadic virus production and limited gene expression. It was not clear if the state of VZV persistence was experimentally reversible and reactivatable. We are not aware of additional publications using this system beyond the first report.

### Human Pluripotent Stem Cell-Derived Neuron Culture Models

Recent advances in generating differentiated human neurons from pluripotent stem cells have widened the choice of systems for modeling VZV neuronal latency. Stem cell models are genetically consistent and expandable, and stem cells can be guided to differentiate toward specific neuronal subtypes, all ideal for current and future applications. Possible drawbacks include that the resulting neuron cultures are heterogeneous to varying degrees, and lack the complexity of human ganglia with its many neuronal and non-neuronal cell interactions. Both human embryonic stem cells (hESC) and induced pluripotent human stem cells (iPSC) have been developed into neuron cultures for VZV study, and differentiation protocols continuously evolve (Tang et al., 2017). At the time of this review, only hESC-derived neurons have permitted an experimental state of VZV genome persistence to be established that can later be reactivated (Markus et al., 2015; Sadaoka et al., 2016).

Several groups have reported the use of iPSC to generate neuron culture models for VZV infection. Lee et al. (2012) derived their own iPSC line from primary human embryonic fibroblasts using the classic retroviral mediated expression of Oct4, Sox2, cMyc, and Klf4. These iPSC were directed to differentiate into neural progenitors using a combination of small molecule inhibitors of SMAD, GSK and Notch signaling, followed by culture with a combination of neuronal growth factors. The resulting heterogeneous neuron population stained 80% positive for neuronal marker βIII tubulin, with 15% co-expressing βIII tubulin along with the sensory neuronal markers BRN3a and peripherin. Electrophysiological activity was also demonstrated in these cells. The neuron cultures were reported to support a productive VZV infection, cell-to-cell spread, and release of infectious virus, but were not shown to model persistence.

Baird and colleagues report use of a commercially available iPSC-derived neuronal precursor that is immediately plated in defined proprietary media to generate “iCell” neurons (Cellular Dynamics International, Inc.) (Baird et al., 2013, 2014b, 2015; Yu et al., 2013). The supplied mixture consists of post-mitotic neuronal subtypes of mainly of GABAergic and glutamatergic neurons that can be cultured longer than 21 days. Cultures are 95% positive for βIII tubulin expression and show characteristic neurite and axonal projections (Yu et al., 2013). Experimental infection with cell-free VZV leads to extensive transcript and protein expression (Baird et al., 2013, 2015; Yu et al., 2013). While initial reports suggested a complete lack of cytopathic effect (Yu et al., 2013), more recent work reported a fully expressed viral transcriptome, virus production and cytopathic effect (Grose et al., 2013; Baird et al., 2014a, b, 2015). The iCell neuron system was used to show the influence of cytokines IL6, IFNγ and type 1 IFNs in blocking virus growth and gene expression (Baird et al., 2015; Como et al., 2018). It is not yet clear if these cells can host a reactivatable, persistent state.

Using hESCs as the starting material permits some level of reproducibility that can be followed by other groups. At the time of this review, three reports detail their use for study of VZV replication, latency and experimental reactivation. While each differs in the process by which neurons are differentiated, the similarities between the models are more important, as they clearly point to the first bone-fide models of VZV reactivation. The first, detailed in several publications from the laboratories of the Goldstein and Kinchington groups, used the WiCell hESC line WA09 (also known as H9; Thomson et al., 1998). When cocultured with the murine stromal PA6 line, renewable neurosphere precursors are generated that can be maintained in a precursor state on Matrigel or in suspension. Seeding onto laminin-coated plates with neurobasal media containing BDNF, NGF and NT3 for a 2-week terminal differentiation process leads to cultures consisting predominantly of neurons with extensive neurite projections. Cultures stained 95% positive for βIII tubulin with very few GFAP-positive cells or Ki67-positive dividing precursors. A fraction stain positive for the sensory neuron markers BRN3a and peripherin, indicating some neuron heterogeneity.

These hESC-neurons were used with fluorescent reporter viruses that permitted live cell monitoring of acute infection, lytic gene expression, cell-to-cell spread, and the absence of protein expression during a modeled latent state. In particular, recombinant VZV expressing GFP tagged to the amino terminus of the ORF66 protein kinase, or to the ORF23 capsid protein have been used. The neuron cultures were fully permissive for VZV infection and spread when initiated by cell-associated infection at the soma or axon, or by high levels of cell-free virus at the soma (Markus et al., 2011). Infecting cells fused to neuron soma and axons, delivering not only virus to the neuron, but also intracellular components of the infecting cell (Grigoryan et al., 2015). This may drive the predominantly lytic infections that result. Electron microscopy established that the neurons were productively infected and extensively coated with capsids packaged with DNA on their cell surface. Infected neurons also released infectious virus into the media, a property not shared with VZV replication in most non-neuronal cultures. The platform has also been used to address neurotropism of VZV recombinants lacking VZV genes, and it was demonstrated that ORF7, a protein important for VZV spread in skin culture (Zhang et al., 2010) and in differentiated SH-SY5Y cell infections (Selariu et al., 2012) was critical for neuronal spread.

**FIGURE 1:**
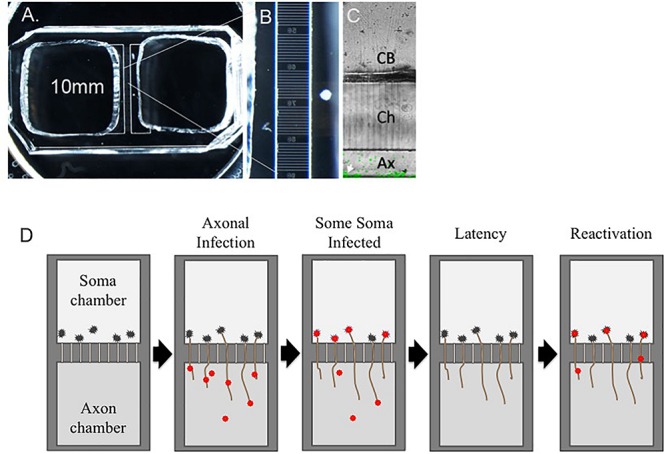
Latent VZV infections established in hESC-neurons in microfluidic devices can be experimentally reactivated. hESC-neurons are infected axonally in microfluidic devices that separate neuronal soma from axons. **(A–C)** Depicts a microfluidic chamber that separates neuronal soma (CB) from axons (Ax). VZV infection is established in the axonal compartment, allowing VZV to be transported through axons into some somata, in which a quiescent infection is established. Upon stimulation with PI3K inhibitor LY294002 and incubation of cultures at 34°C, virus in some latently infected neurons is reactivated and productive infection is re-established. Ch = microfluidic channels that connect the soma and axon compartments. **(D)** A diagrammatic representation shows the process of axonal infection of neurons with VZV, establishment of latency, and reactivation.

Culture of neurons in microfluidic chamber devices (Figure 1) permitted the separation of neuron soma from axons. When axons were infected with cell-associated VZV, lytic, spreading infections developed on the soma side (Markus et al., 2011). These devices also permitted the first live cell visualization of VZV capsid retrograde transport inside axons using a fluorescent ORF23 capsid protein reporter virus; ORF23 encodes the abundant capsid protein homologous to HSV VP26 (Grigoryan et al., 2012). The motion of VZV capsids was neither continuous nor exclusively retrograde, but capsids migrated toward the neuronal soma at a rate similar to that of the neurotrophic alphaherpesviruses HSV-1 and PRV (Smith et al., 2001; Antinone and Smith, 2010).

The first reactivatable latent infection was demonstrated in this hESC-neuron fluorescent-VZV system (Markus et al., 2015). Persistence was established by infection of neuron cultures in microfluidic devices, either at the soma with cell-free virus in the presence of the antiviral acyclovir, or by axonal infection without use of antivirals. The antiviral approach has been successfully employed to model HSV latency *in vitro* to select against initiating lytic events (Hunsperger and Wilcox, 2003; Kobayashi et al., 2012). It is now recognized that neurons are more sensitive to cell-free VZV infection than other cell types (Sadaoka et al., 2018). Using VZV66GFP, lytic infection in the cultures could be differentiated from non-lytic infected cultures in real time. It was demonstrated that GFP negative cultures still contained VZV genomes in neuronal nuclear puncta, using both qPCR and fluorescent *in situ* hybridization (FISH). FISH showed the viral genome to be localized to single or dual nuclear puncta, similar to that recently reported for HSV latent infections (Catez et al., 2014). Surprisingly, transcriptional analyses by RNA-seq revealed a widespread general suppression of all lytic gene transcripts, but with some ORFs showing relatively higher levels as compared to a lytic infection, particularly ORFs in the repeat region. VLT RNA had not yet been described and was not assessed.

Most importantly, it was demonstrated that disruption of NGF signaling resulted a renewal of GFP positivity and a spreading infection, indicating renewed lytic replication after a long period of GFP absence (Markus et al., 2015). Continuous NGF signaling is required to maintain HSV latency in model systems (Camarena et al., 2010). VZV reactivation, indicated by renewed GFP expression, was achieved by growth factor withdrawal, the inhibition of PI3-Kinase signaling with the inhibitor LY7294002, or the treatment of latent cultures with the type 1 histone deacetylase-inhibitor sodium butyrate. GFP positivity correlated with expression of lytic genes, increased VZV DNA, an increase in the number of puncta by FISH, and renewed virus production capable of infecting ARPE19 cells. Intriguingly, incubation at reduced temperature increased the reactivation frequency and reduced inhibitor toxicity.

The microfluidic chamber system was then used to establish latency without antivirals using axon only infection with cell-free virus. This is more physiologically relevant and reflects infection that would occur in establishing latency from skin vesicles. It had been previously shown that HSV axonal infection preferentially results in a latent infection, most likely due to poor delivery of virion tegument transactivators of lytic infection down the axon to the neuronal nucleus (Hafezi et al., 2012). Even high MOI infections with cell-free VZV failed to initiate GFP-positive (lytic) infected soma in the cell-body chamber, but since soma contained VZV genomes, latency must have been established. Again, reactivation was demonstrated by renewed GFP expression in previously GFP-negative soma chambers stimulated by the PI3K inhibitor. Taken together, these experiments established use of hESC-derived neurons to model an experimentally reactivatable latent VZV infection.

The hESC experimental model was more recently used to explore VZV encoded sncRNAs. While previous studies had not detected miRNA in human trigeminal ganglia latently infected with VZV (Umbach et al., 2009), short miRNA-length RNAs were identified using NGS methods in fibroblasts and hESC-derived neurons lytic infected with VZV (Markus et al., 2017). Unconventional bioinformatic analyses predicted 24 VZV encoded small non-coding RNAs (VZVsncRNA) in NGS reads of productively infected neurons. The presence of one VZVsncRNA was confirmed using stem-loop rt-qPCR, and one was detected in latently infected neurons. These studies established that VZV, like other herpesviruses, makes use of small RNAs that may regulate infection. As noted above, two sncRNAs map to the introns of the VLT transcript (Markus et al., 2017). This work is an area that will likely be expanded in the near future in addressing the contribution of VZVsncRNA to the molecular mechanisms underlying VZV latency.

A second group reported a similar system to host a VZV reactivatable latent state (Sadaoka et al., 2016). Their neuron platform was differentiated from commercially available hESC-derived neural stem cells (Life Technologies, Inc.), originally differentiated from the same WA09 hESC line used by Markus et al. (2015). The neuron cultures were predominantly βIII tubulin positive, with a fraction of cells also expressing the sensory neuron markers BRN3a and peripherin, as seen by Markus et al. (2015). Only very low levels of cells stained with GFAP, a marker of radial glia, astrocytes and satellite cells. There was no positive staining for microglia or oligodendrocytes in the cultures. Their model predominantly relied on a microfluidic chamber system to separate axons and soma, followed by cell-free VZV infection of the axon or somal compartments. Like Markus et al. (2015), they reported the initiation of lytic events when neuronal soma were infected with cell-free VZV (strain Parent of Oka or POka) at high MOI, but also found that low MOI cell-free VZV infections at the soma could result in VZV DNA positive cultures showing no detectable lytic growth or cytopathic effect. In agreement with Markus et al. (2015), cell-free infection of compartmentalized neurons was not productive, but delivered VZV DNA to the somal compartments. They also saw very low levels of transcription of all VZV ORFs by RNAseq, indicating that most lytic gene expression was silenced. Viral DNA could be detected for up to 70 dpi in the neurons without evidence of replication, and the retained VZV genomes were predominantly circular or endless, as seen for other latent herpesviruses and for latent VZV in human cadaver ganglia (Clarke et al., 1995). This contrasted with the predominantly linear VZV genomes seen in lytic infected cultures. Importantly, reactivation was demonstrated as renewed lytic infections in approximately 25% of latently infected cultures when NGF was depleted using antibodies. Reactivation resulted in spreading productive infections and virus capable of infecting MRC5 cells.

Another intriguing study compared the parent and vaccine strains of Oka for the establishment of latency and reactivation from it. The genetically heterogeneous attenuated vaccine retains the ability to go latent and has caused zoster in vaccinated individuals, albeit at a greatly reduced level compared to wild type strains (see Breuer, 2018 for recent review). The authors found that the vaccine strain could still establish latency in the hESC-neuron model at levels similar to the wild type parent Oka strain, and with a level of genome heterogeneity seen in the vaccine. However, the vaccine virus expressed a considerably reduced level of viral transcripts and was an average of fivefold less efficient at reactivating after the experimental stimulus of NGF removal. Furthermore, the genomic diversity was considerably reduced in the *in vitro* reactivated virus, indicating either there was some clonal selection of genomes more able to reactivate over others in the heterogeneous vaccine strain, or alternatively, there was a stochastic process in which a very low number of genomes are induced to reactivate. This bottleneck may explain the restricted genome diversity seen in breakthrough lesions after vaccine inoculation (Depledge et al., 2014).

A comparison of the two models is warranted. Both systems (Markus et al., 2015; Sadaoka et al., 2016) used axonal infection in microfluidic devices to set up VZV latency without use of antivirals. Both groups showed that initiation of infection at the soma compartment with cell-free virus resulted in lytic infections. Markus et al. (2015) also set up latency using ACV to inhibit lytic infections in non-compartmentalized directly infected neuron cultures. ACV has been used routinely to block HSV lytic infections in several neuronal model systems to favor establishing a latent state. The use of ACV may be problematic, because any initiated lytic infection will express the deoxypyrimidine kinase and result in the formation of ACV-PPP, which can then become incorporated into DNA and act as a chain terminator. This may render viral genomes as damaged and less able and/or likely to reactivate. As such, latent genomes seen in ACV-treated cultures are probably a mixture of undamaged genomes from infections that enter latency immediately after infection, and ACV-damaged genomes maintained after an initiated lytic infection that becomes repressed after blockade of DNA replication. A minor difference between the two studies is temperature. Sadaoka et al. (2016) reactivated neuron cultures at 37°C, while Markus et al. (2015) reported efficient reactivation at 34°C.

The model of Sadaoka et al. (2016) was more recently exploited to examine the role of c-Jun N-terminal kinase (JNK) in neuronal VZV infection. It was previously indicated that VZV modulates the JNK pathway in human foreskin fibroblasts, increasing JNK and active phospho-JNK. JNK inhibition negatively impacts VZV replication (Zapata et al., 2007), although Rahaus et al. (2004) reported the opposite effect in MeWo cells and suggested blocking JNK was pro-viral. Kurapati et al. (2017) reassessed the role of JNK in VZV infection of neurons, reporting that VZV induced JNK activation and JNK phosphorylation in neuron cultures. The group exploited the microfluidic chamber system to establish persistent VZV infections by axonal infection, and found that chemical inhibition of JNK reduced the efficiency of viral reactivation by up to fourfold. This suggests the potential use of JNK inhibition as a reactivation inhibition strategy.

It is likely that the hESC-neuron platforms will undergo continuous development in the near future. One such development was included in the work by Kurapati et al. (2017), in which they generated cultures highly enriched for sensory neuron phenotypes. These cultures were generated from a genetically modified hESC Wa09 line with a GFP reporter for the neural crest marker SOX10 that allowed selection by flow sorting (Lee et al., 2010). Application of inhibitors of SMAD and canonical WNT signaling to cultures of sorted NC-like cells resulted in efficient (95%) generation of sensory neurons expressing BRN3a and peripherin (Mica et al., 2013). They showed the sensory neuron cultures were fully permissive for VZV and that the JNK inhibitor reduced VZV replication.

## Questions and Perspectives on the Future of Modeling VZV Latency and Reactivation

(1) The differentiation of hESC into specific subtypes of neurons and lineages is a field of intense interest and continuous development that will impact not only the study of the VZV latent state, but also that of HSV-1 and HSV-2. Evidence is accumulating that suggests some neuronal subtypes are more restrictive for virus replication than others. Work from the human fetal ganglionic implant-SCID-hu model has suggested that peripherin-positive nociceptors are more susceptible to VZV infection as compared to mechanoreceptor neurons marked by the RT97 antibody, which exhibit some restriction to VZV infection (Zerboni and Arvin, 2015). Advances in this area may enable us to address whether the more restrictive neurons host a VZV genome that can eventually reactivate. It may also allow study of the possibility that different subtypes of neurons reactivate in response to different stimuli. While studies have not yet been detailed, we consider it likely that VZV is capable of establishing genomes in many more subtypes of neurons than HSV. HSV does not generally exhibit a viremia and predominantly establishes latency through the infection of axons that infiltrate the periphery and site of infection. This contrasts to VZV, which likely accesses the ganglia by both retrograde axonal transport from the periphery and by direct infection from ganglia-infiltrating VZV infected immune cells. Neurons in ganglia that do not innervate the periphery may be responsible for VZV clinical presentations other than classic zoster, such as zoster without rash (sine herpete) and the numerous enteric, neurological and vascular conditions associated with VZV reactivation. The types of neurons that host VZV genomes and those supplying reactivation is a direction ripe for further exploration.
(2) The discovery of VZV VLT RNA is a recent development that is yet to be included in models of VZV latency, *in vivo* or *in vitro* (Depledge et al., 2018a). The conserved features of VLT with latency transcripts of other neuronal alphaherpesviruses is highly suggestive of important roles that have been functionally retained as alphaherpesviruses have diverged and evolved. In spite of extensive research on the better-studied HSV-1 system, what those conserved functions are is not yet clear. With some further development, we should be poised to determine how the VLT and/or transcripts mapping to ORF63 contribute in the models of latency and reactivation, both *in vivo* and *in vitro* (Depledge et al., 2018a). Are both RNAs expressed in all or only a fraction of VZV latently infected neurons? Do these represent expression in different neuronal subtypes, or is expression of the two transcripts linked? What is the pattern of their expression? It remains a possibility that either RNA is a product of the very early post mortem-induced transcription program, as even minimal PMIs cannot be avoided.
(3) VZV genetic model systems can now be used to probe possible functions of VLT and other genes in the onset of human neuronal infection, latency and reactivation. VZV is readily genetically manipulated through the use of BAC and cosmid systems (Osterrieder et al., 2003; Tischer et al., 2007) and viruses from BACs and cosmids go latent and reactivate in the hESC model platforms (Markus et al., 2015). It should be straightforward to generate VZV VLT mutants, although it may still be quite challenging to evaluate how latency and reactivation is influenced by VLT mutation or deletion. The functions of the more extensively studied HSV LAT are still quite controversial. LAT has long been speculated to regulate the multifunctional transactivator protein HSV ICP0 (Wagner et al., 1988a, b), a key factor in the establishment of lytic infections, and VLT may act the same on ORF61. The HSV-1 LAT RNA seen during latency is a stable intron from several splice variants of a difficult-to-detect primary 8.3 kb transcript, which accumulates in the nucleus of latently infected neurons (Wechsler et al., 1988; Zwaagstra et al., 1990). HSV-1 LAT has been implicated to influence the regulation of chromatin (Bloom et al., 2010), express miRNAs that influence gene expression (Umbach et al., 2008), regulate apoptosis (Perng et al., 2000), and impact the survival of latently infected neurons (Thompson and Sawtell, 2001) and the efficiency of reactivation (Watson et al., 2018). However, the fact that HSV-1 lacking LAT can still establish persistence and reactivate in all current *in vivo* animal models does imply that LAT roles are not essential. We will likely soon see whether VLT is required for establishing latency and reactivation in the human neuron systems that are now available. It is likely that HSV-1 LAT function will be re-assessed in these human neuron systems.
(4) The *in vitro* human neuron platforms in use do have some limitations that may require a further study of the *in vivo* model systems of persistence. The *in vitro* systems are not yet sufficiently developed to permit studies of how latent infections interact with the adaptive immune system. This is a particularly important issue, given that declining adaptive cellular immunity is a major predictor of zoster incidence, and that vaccination-mediated boosting of immunity reduces rates of zoster. The contribution of adaptive immunity will probably need an *in vivo* system, and the guinea pig model appears to be the most likely permissive *in vivo* system. However, while the guinea pig model has been reported to enter a latent state that is experimentally reactivatable (Gershon and Gershon, 2018), reactivation is not yet fully documented, characterized or optimized. Alternatively, some aspects of the influence of acquired immunity on reactivation may come from further *in vitro* model development, such as by developing MHC-compatible iPSC-derived neuron cultures from the same host in which T cells have been isolated. Alternatively, hESC could potentially be modified to be MHC compatible with a donor immune cell population.

Glia surround and are believed to interact with neurons in both the central and peripheral nervous systems. Thus, satellite glial cells (SGC) and Schwann cells in peripheral ganglia may play a role in establishment and/or maintenance of latent VZV infections. Evidence from the SCID-hu mouse xenografted with fetal human DRG suggests that SGC are infected by VZV and fuse with neighboring neurons (Zerboni and Arvin, 2011). Schwann cells have been generated from hESC and were shown to myelinate human and chick embryo neurons (Ziegler et al., 2011). SGCs have also been implicated in the modulation of neuropathic pain (recently reviewed in Fan et al., 2019). It is therefore likely that additional insights into VZV latency may be obtained by addition of glial cells to the *in vitro* human neuron model for VZV infection.

## Conclusion

Advances in the development of *in vitro* human neuron systems for modeling VZV latency and reactivation and the recent discovery of the VZV latency-associated transcript have set the stage for a new era in resolving this perplexing persistent state.

## Author Contributions

As this manuscript is a review, all authors were involved in writing and editing the manuscript. There were no new experiments included in this work.

## Conflict of Interest Statement

The authors declare that the research was conducted in the absence of any commercial or financial relationships that could be construed as a potential conflict of interest.
